# Hutchinson-Gilford progeria syndrome accompanied by severe skeletal abnormalities in two Chinese siblings: two case reports

**DOI:** 10.1186/1752-1947-7-63

**Published:** 2013-03-08

**Authors:** Zhimin Xiong, Yanmei Lu, Jinjie Xue, Sanchuan Luo, Xiaojuan Xu, Lusi Zhang, Hao Peng, Wei Li, Dengming Chen, Zhengmao Hu, Kun Xia

**Affiliations:** 1 State Key Laboratory of Medical Genetics, Central South University, Changsha, Hunan, China; 2 School of Biological Science and Technology, Central South University, Changsha, Hunan, China; 3 Key Laboratory of Medical Information Research, Central South University, Changsha, Hunan, China; 4 Department of Radiology, Xiangya Hospital, Central South University, Changsha, Hunan, China

## Abstract

**Introduction:**

Hutchinson-Gilford progeria syndrome is a rare pediatric genetic syndrome with an incidence of one per eight million live births. The disorder is characterized by premature aging, generally leading to death due to myocardial infarction or stroke at approximately 13.4 years of age. The genetic diagnosis and special clinical manifestation in two Han Chinese siblings observed at our clinic for genetic counseling are described in this report. We screened the *LMNA* gene in these two siblings as well as in their unaffected parents. A homozygous mutation R527C was identified in the affected siblings, and both parents were heterozygous for this variant.

**Case presentation:**

In case 1, the elder 10-year-old female sibling showed the classic physical and radiological changes of Hutchinson-Gilford progeria syndrome in addition to a considerable overlap with the phenotype of mandibuloacral dysplasia.

In case 2, the younger male sibling had begun to show some early physical changes at age six months.

**Conclusion:**

The phenotypic findings in the patients we describe here widen the clinical spectrum of Hutchinson-Gilford progeria syndrome symptoms, providing further recognition of the phenotypic range of *LMNA*-associated diseases.

## Introduction

Hutchinson-Gilford progeria syndrome (HGPS; OMIM:176670) is an extremely rare but devastating disorder characterized by extremely short stature, low body weight, early hair loss, lipodystrophy, scleroderma, decreased joint mobility, osteolysis and facial features that resemble those of aged persons [1,2]. The incidence of HGPS is estimated to be one in every eight million live births [2]. The diagnosis is usually made by two years of age. About 144 cases have been described in the literature worldwide [1,3]. The pattern of inheritance is uncertain, though both autosomal dominant and autosomal recessive modes have been proposed [4,5].

Recent genetic advances have identified *LMNA* as a causative gene of HGPS. *LMNA* encodes lamins A and C, which are the main components of intermediate filamentous lamina, function as a structural support, and are essential for DNA replication and mRNA transcription. In addition, these proteins play a role in gene regulation and many signal transduction pathways [6]. Here, we report the case of two Han Chinese siblings who harbor a homozygous change that predicts an R527C mutation, similar to that in another case that has been recently reported [3].

## Case presentations

### Case 1

Our patient was a 10-year-old Han Chinese girl, observed at our clinic for genetic counseling, who was the product of an unremarkable pregnancy and delivery. She did not exhibit any abnormal symptoms until the age of five months, when she developed skin thickening over the extensor surface of her joints followed by progressive difficulty in extending her fingers. Shortly thereafter, she began to lose her hair. Her younger brother has a physical appearance similar to his sister. The living blood relatives of the parents are negative for the phenotype, and the parents have no blood relationship.

On examination, the girl was found to have an extremely short stature (height 93.5cm; weight 6.5kg) and a head circumference of 47.3cm. She was bald, had no eyebrows or eyelashes, and had prominent scalp veins. She also had prominent eyes, a small face and large head, a convex nasal ridge, micrognathia and crowded teeth, narrow shoulders and a horse-riding stance, and a high-pitched voice. There was a generalized paucity of subcutaneous fat. Her atrophic skin was thin, dry and taut, and was marked with diffusely scattered hyperpigmented foci. There was a fixed flexion deformity in all of her fingers. Her anterior fontanel was open (1.5cm×1.5cm).

**Figure 1:**
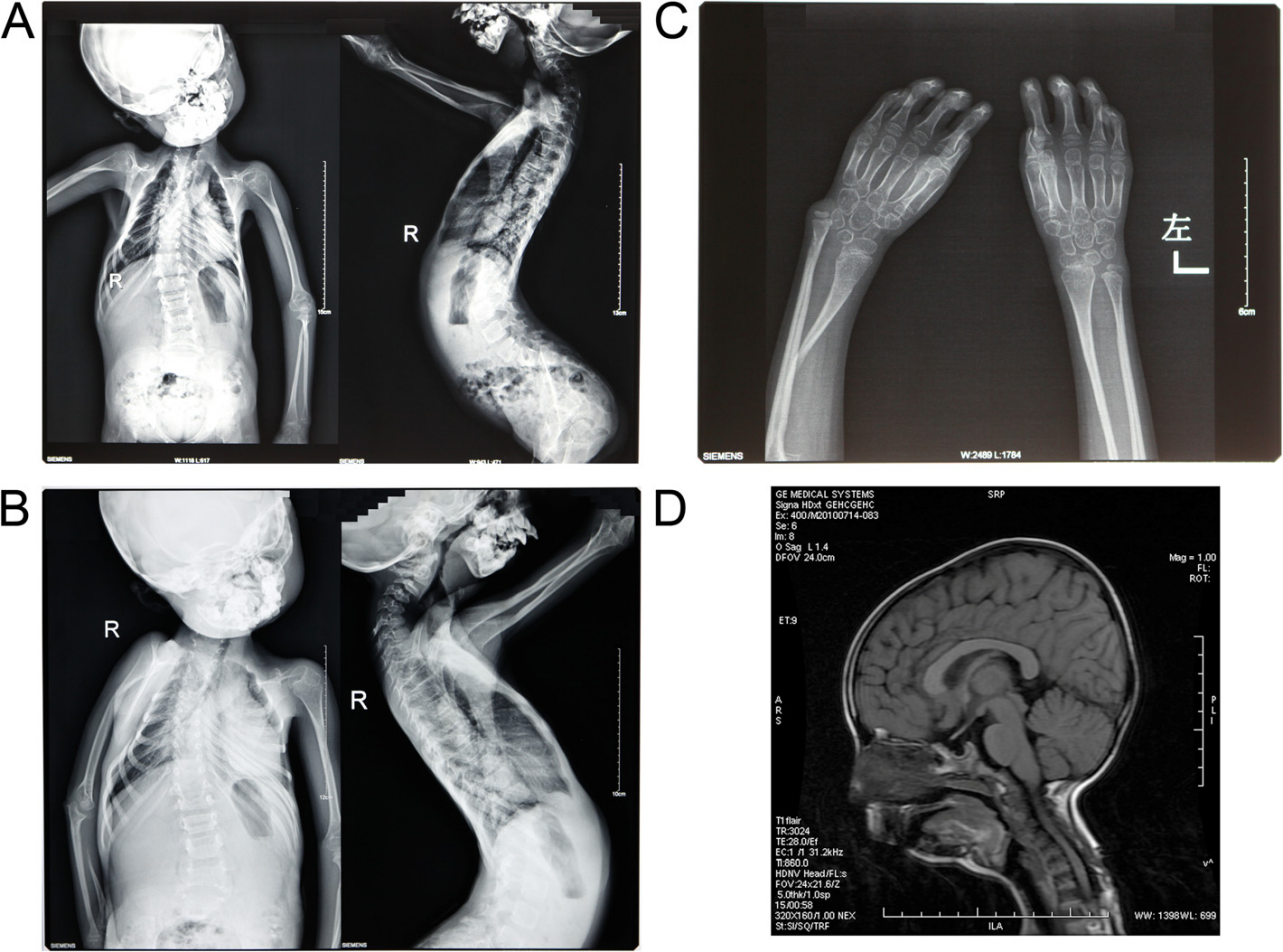
**Radiography and magnetic resonance imaging features in case 1. **(**A,B**) The elder sibling showed generalized and marked osteoporosis with relative expansion of the metaphyseal areas of the bones, completely absent clavicles, partial osteolysis of the bilateral posterior segments of the first to fourth ribs, absence of the costal head of the right 12th rib; severescoliotic deformities, complete osteolysis and resorption of the superior fragment of both radii, a dislocation of the inferior segment of the right ulnoradial joint, and an old, healed fracture of the interior segment of the right ulna. (**C**) Fixed flexion deformities of the phalanges in the elder sibling with absence of the distal phalanges of the second to fifth fingers. (**D**) Magnetic resonance imaging features of the elder sibling showing a small pituitary gland with a concavity of the superior border (with a height of about 1mm).

Radiography revealed generalized and marked osteoporosis with relative expansion of the metaphyseal areas of her bones, a large cranium and a relatively small viscerocranium. Both her maxillae and mandibles were hypoplastic with crowded teeth, malocclusion and protrusion of her upper teeth. Both of her clavicles were completely absent, the bilateral posterior segments of her first to fourth ribs were partially osteolytic, and the costal head of her right 12^th^ rib was absent. She had severe scoliotic deformities: her cervical segment showed fixed flexion deformities and her fifth lumbar segment had a slightly forward olisthe. There was complete osteolysis and resorption of the superior fragment of both radii, the inferior segment of her right ulnoradial joint had a dislocation, and there was an old, healed fracture of the inside of her right ulna. There were fixed flexion deformities of her phalanges: the middle phalanges of her fingers became more slender, and the distal phalanges of the second to fifth fingers were absent (Figure 1A-C).

**Figure 2:**
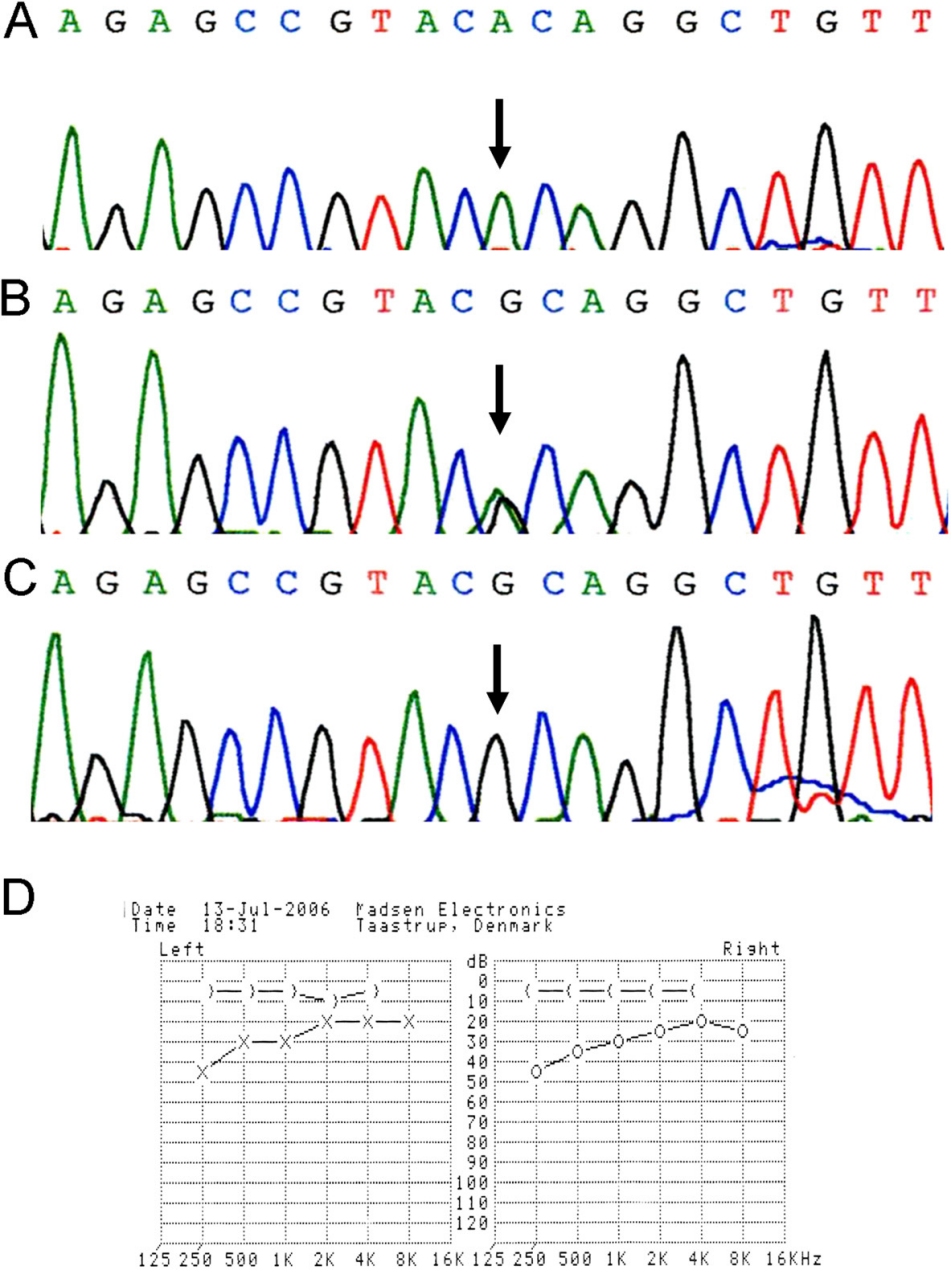
***LMNA *R527C in the two siblings and their parents and audiologic testing in case 1.** (**A**) Homozygous mutation of R527C in the two siblings. (**B**) Heterozygous mutation of R527C in parents. (**C**) Normal control sequence. (**D**) Audiologic testing showing that the elder sibling had a conductive hearing loss in the low-frequency range (250Hz to 500Hz) in both ears.

Magnetic resonance imaging revealed normal brain tissue with mastoiditis of both middle ears. Our patient had a small pituitary gland with a concavity of the superior border (height about 1mm. Figure 1D). Audiologic testing revealed conductive hearing loss in the low-frequency range (250Hz to 500Hz) in both ears. Echocardiography revealed left ventricular hypertrophy with a mild regurgitation of the tricuspid valve and pulmonary valve (Figure 2D). Carotid ultrasonography detected a narrow carotid artery intima-media thickness.

Laboratory results of hepatic function and her levels of blood calcium, blood phosphonium, blood free tri-iodothyronine, free thyroxin, thyroid-stimulating hormone, serum cholesterol, triglyceride, lipoproteins, fast blood glucose, prothrombin time and hemoglobin were all within the normal ranges. Her renal function showed a low level of creatinine (29.6μmol/L; normal range 53 to 132.6μmol/L). Her platelet count was elevated.

### Case 2

The 18-month-old younger brother of our first patient had begun to lose his hair at the age of six months. He had prominent eyes and open fontanels. His scalp veins were prominently visible. His skin was thickened and showed a striking resemblance to that of his sister. Radiography revealed an attenuation of the skull board, widely patent sutures, and the absence of left and right lateral clavicles.

A chromosome analysis (resolution, 500 to 600 bands) revealed that the siblings had a normal karyotype ((46,XX) and (46,XY) respectively). We then sequenced the entire coding region of the *LMNA* gene for the affected siblings and their unaffected parents. Mutation analysis revealed a homozygous mutation 1579C>T, which predicts R527C, in the two siblings; both their parents were heterozygous for this variant. By contrast, no variants of this specific mutation were found in the 100 unrelated control individuals (Figure 2A-C).

## Discussion

Our patient in case 1 exhibited the typical phenotype of HGPS, showing the initial symptoms in the first year of life, severe growth deficiency, expressed lipodystrophy, expressed hair loss, and severe osteolysis of the acra, clavicles, mandible and viscerocranium. However, her severe skeletal abnormalities, which were similar to those of other sibling sets described by Monu *et al*. in 1990 [7], have a considerable overlap with the phenotype of mandibuloacral dysplasia, a disorder mainly characterized by skeletal malformations. In addition, there were other unusual features in our patient. First, the scoliotic deformities, such as the olisthy of her lumbar segments, were much more severe than in the previously reported cases. Second, the degree of osteolysis and resorption of her ribs was much greater. Third, the dislocation of the inferior segment of her right ulnoradial joint and the low level of creatinine that we observed in this patient has, to the best of our knowledge, not been reported before. Fourth, the results of the magnetic resonance imaging revealed a pituitary gland small for our patient’s age.

The mode of inheritance of the disease remains largely speculative for now because the patients affected cannot reproduce. Although recurrent heterozygous *de novo* mutations of *LMNA* strongly support a mode of inheritance of HGPS as a sporadic dominant disorder, autosomal recessive modes of inheritance have been proposed in siblings and blood relatives [5].

Most of the classic cases of HGPS are reported to be caused by a single base substitution 1824C>T in *LMNA*, which activates a cryptic splicing site [8]. Here we identified a homozygous mutation c.1579C>T *LMNA* in two siblings, which predicts an R527C mutation. Results of the clinical examination of the parents, who were heterozygous for this mutation, were normal. We conclude that the inheritance of the phenotype in our patients is autosomal recessive. The R527C mutation was first described in a 28-year-old female compound heterozygote patient with R471C, affected with mandibuloacral dysplasia [9]. The homozygous mutation R527C has been reported recently in two Chinese siblings [3] whose phenotype was categorized as atypical HGPS with severe skeletal abnormalities.

Mutations in *LMNA* have been identified as the cause of at least 12 different inherited disorders [10,11], known as laminopathies, which have significant phenotypic overlap [12]. However, it remains unclear how different mutations in the *LMNA* gene cause such a variety of phenotypes. For instance, at the same coding site 527, three different substitutions have been reported to cause three different diseases: R527P causes autosomal dominant Emery-Dreifuss muscular dystrophy [13], whereas R527H is associated with mandibuloacral dysplasia [14], as is the compound heterozygote R527C/R471C [9]. The homozygote R527C causes either atypical [3] or typical HGPS.

The encoding products of *LMNA* are part of an intermediate filament family of proteins that are composed of an N-terminal end, a coiled-coil rod domain and a globular C-terminal tail domain [15]. The R527C mutation described here resides in the C-terminal tail domain of the protein. The result of the substitution of a basic amino acid (arginine) to a neutral one (cysteine) in this location would disrupt the surface structure of lamins, thus causing the severe diseases observed.

## Conclusion

The phenotypic findings in the patients we described here widen the clinical spectrum of HGPS symptoms, and provide further recognition of the phenotypic range of *LMNA*-associated diseases.

## Consent

Written informed consent was obtained from the patients’ mother for publication of this case series and accompanying images. A copy of the written consent is available for review by the Editor-in-Chief of this journal.

## Competing interest

The authors declare that they have no competing interests.

## Authors’ contributions

XZM analyzed and interpreted the patient data and was a major contributor in writing the manuscript. XJJ and CDM interpreted the patient data regarding X-ray and magnetic resonance imaging. LYM and LSC performed the genetic analysis. XXJ, ZLS, PH, LW and HZM performed laboratory examinations. XK communicated with the patients and revised the manuscript. All authors read and approved the final manuscript.
